# Defining metrics of visual acuity from theoretical models of observers

**DOI:** 10.1167/jov.24.4.14

**Published:** 2024-04-16

**Authors:** Charles-Edouard Leroux, Conor Leahy, Justine Dupuis, Christophe Fontvieille, Fabrice Bardin

**Affiliations:** 1 Laboratoire MIPA, Université de Nîmes, Nîmes, France; 2 Carl Zeiss Meditec, Inc., Dublin, California, USA; 3 Laboratoire MIPA, Université de Nîmes, Nîmes, France; 4 Laboratoire MIPA, Université de Nîmes, Nîmes, France; 5 Laboratoire MIPA, Université de Nîmes, Nîmes, France

**Keywords:** Fourier optics, models of ideal observer, monochromatic aberrations, metrics of visual image quality

## Abstract

Many experimental studies show that metrics of visual image quality can predict changes in visual acuity due to optical aberrations. Here we use statistical decision theory and Fourier optics formalism to demonstrate that two metrics known in the field of vision sciences are approximations of two different theoretical models of linear observers. The theory defines metrics of visual acuity to potentially predict changes in visual acuity due to optical aberrations, without needing a posteriori scale or offset. We illustrate our approach with experiments, using combinations of defocus and spherical aberration, and pure coma.

## Introduction

Relating wavefront measurements to visual performance is a basic question in the field of spatial vision, with major implications for clinical applications of aberrometry. Among all kinds of proposed approaches, using a metric of visual image quality (Cheng, Thibos, & Bradley, 2003; Chen, Singer, Guirao, Porter, & Williams, 2005), that is, a single number directly computed from the wavefront, has emerged as the most practical approach to account for experimental measurements of visual performance (Marsack, Thibos, & Applegate, 2004; Cheng, Bradley, & Thibos, 2004) and to estimate an objective refraction based on aberration measurements (Guirao & Williams, 2003; Martin, Vasudevan, Himebaugh, Bradley, & Thibos, 2011; Kilintari, Pallikaris, Tsiklis, & Ginis, 2010). Several metrics relate to the Strehl ratio, with different approaches to take account of the neural processing of retinal images (Thibos, Hong, Bradley, & Applegate, 2004). The visual Strehl computed in the spatial domain (VSX) quantifies the fraction of the eye’s point spread function (PSF) that overlaps with a neural weighting function. The VSX metric has been shown to be reliable for objective refraction (Hastings, Marsack, Nguyen, Cheng, & Applegate, 2017) and can quantify optical quality after refraction (Hastings, Marsack, Thibos, & Applegate, 2018). The visual Strehl based on the optical transfer function (VSOTF) quantifies the peak of PSF after taking account of the neural contrast loss that is modeled by the neural transfer function (NTF). The VSOTF metric accounts for measurements of the eye’s depth of focus (Zheleznyak, Sabesan, Oh, MacRae, & Yoon, 2013; Zheleznyak, Jung, & Yoon, 2014; Yi, Iskander, & Collins, 2011) and the effect of optical aberrations on the accommodative response (Buehren & Collins, 2006). The visual Strehl ratio can also be computed from the modulation transfer function (VSMTF metric). The VSMTF metric predicts the accommodative response of the eye in the presence of aberrations (Tarrant, Roorda, & Wildsoet, 2010; López-Gil et al., 2013).

For experiments in which the subject serves as their own control, metrics of visual Strehl (VSX, VSOTF, and VSMTF) correlate remarkably well with changes in visual acuity due to optical aberrations (Marsack et al., 2004). These metrics are also robust to the amplitude of aberration and pupil size (Ravikumar, Sarver, & Applegate, 2012), and can predict the effect of normal or keratoconic aberrations (Ravikumar, Marsack, Bedell, Shi, & Applegate, 2013). The correlation between metrics of visual image quality and absolute visual performance depends on experimental conditions such as the overall visual performance of the population (Villegas, Alcón, & Artal, 2008), light level, and optotype contrast (Applegate, Marsack, & Thibos, 2006). Light scattering and subject-dependent neural sensitivity are also fundamental aspects of the visual system that may lower the predictive ability of metrics of visual image quality in a clinical study (Bühren et al., 2009). Monte Carlo simulations of the subject performing the visual test have been used to model visual performance, as they make it possible to analyze the effect of the test protocol, the subject’s strategy to identify optotypes, and the neural and optical properties of their visual system (Nestares, Navarro, & Antona, 2003; Dalimier, Pailos, Rivera, & Navarro, 2009; Watson & Ahumada, 2005; Watson & Ahumada, 2008).

In an attempt to bypass Monte Carlo simulations but still model the measurement protocol with great detail, Dalimier and Dainty (2008) used statistical decision theory to predict ratios (with/without aberrations) of contrast sensitivity measurements. They computed ratios of data separability using simulated visual images of the actual test optotypes. Similarly, Watson and Ahumada (2008) introduced an acuity metric to bypass Monte Carlo simulations. This acuity metric slightly differs from the concept of metrics of visual image quality, as commonly defined by the community, because it is computed using the set of simulated visual images and the corresponding templates that are used by the observer for letter identification. The main advantage is to predict ratios (with/without aberrations) of visual performance measurements without needing a posteriori scale or offset. In our previous work, we reckoned that the Dalimier and Dainty model could be further simplified using the “small letter approximation” to define a model-based metric of contrast sensitivity, *M*, which we computed directly from optical aberrations without actually simulating the visual images (Leroux, Fontvieille, Leahy, Marc, & Bardin, 2022). We described this metric as model based, as it inherited from the Dalimier and Dainty model. In this work, we choose as a starting point to adapt the Dalimier and Dainty model to visual acuity and use the small letter approximation. The Dalimier and Dainty model is based on a model of an ideal observer, and we also introduce a more realistic model of a “real observer” to define a second model-based metric of visual acuity. The two model-based metrics are compared to experimental measurements of letters lost with combinations of defocus and spherical aberrations, as well as coma. We demonstrate in this work that metrics of visual image quality can be defined from rigorous models and can be customized to the experimental conditions to predict visual acuity accurately.

## Theory of model-based metrics of visual acuity

### The classification task of theoretical observers

In the framework of statistical decision theory, we model measurements of visual acuity as a classification task, for which the subject is asked to classify optotypes in well-known *K* classes, for example, a known set of Sloan letters. We model the observed data *i*(*x*, *y*) as visual images *I*_*k*, *a*_(*x*, *y*) (*k* = 1 to *K* indexes the letter and *a* is the angular extent of the letter gap), with added independent and identically distributed Gaussian noise (*n*(*x*, *y*), of variance σ^2^) of physiological (mostly neural) origins.

(1) $$i\left(x,y\right)=I_{k,a}\left(x,y\right)+n\left(x,y\right)$$

An observer is a theoretical model of the subject’s own “algorithm” that processes the observed data and classifies it in one of the *K* classes. Among the numerous observers, the ones that process data linearly are interesting models for their mathematical simplicity. To simplify a bit further the classification problem, we first limit our analysis to the binary classification problem (*K* = 2).

### Performance analysis of two linear observers in a binary classification problem

In a binary classification problem, a linear observer computes a scalar t lin , known as the test statistic, as the scalar product of the data and a model template known as the discriminant *T*(*x*, *y*) (Barrett & Myers, 2003, p. 811):

(2) $$t_{\mathrm{lin}}=\int \;\;\;\int i\left(x,y\right)T\left(x,y\right)dxdy$$

The observer chooses between the two classes by comparing t lin  to a threshold.

Among all kinds of linear observers, the ideal linear observer (known as the Hotelling observer) has full knowledge of the expected visual images *I*_*k*, *a*_(*x*, *y*) and uses their difference as the discriminant. The ideal observer computes the following test statistic:

(3) $$t^{*}=\int \;\;\;\int i\left(x,y\right)\left(I_{1,a}\left(x,y\right)-I_{2,a}\left(x,y\right)\right)dxdy$$

t* is the test statistic of the ideal observer. It is given by Equation 3 because of our hypothesis of independent and identically distributed Gaussian noise (Barrett & Myers, 2003, p. 836). The ideal observer makes the best use of the available information in the observed data to perform the classification efficiently: The performance of the ideal observer is only limited by noise. In the presence of optical aberrations, the ideal observer uses the aberrated visual images as templates to compute the discriminant. What makes the observer ideal is its ability to use templates that perfectly match the noise-free data *I*_1, *a*_(*x*, *y*) and *I*_2, *a*_(*x*, *y*). The ideal observer was found to predict the effect of optical aberrations on contrast sensitivity measurement with relatively large optotypes: Landolt C with a 3-arcminute gap (Dalimier, Dainty, & Barbur, 2008; Dalimier & Dainty, 2008) and Sloan letters with 2-arcminute gaps (Leroux et al., 2023). In comparison to other theoretical observers, the ideal observer will predict better visual acuity, especially when visual images are strongly altered by optical aberrations. In these conditions, which correspond to combinations of small letter and high amplitude of aberration, the ideal observer will also become less realistic, as most subjects may not be able to use the aberrated alphabet as image templates.

Watson and Ahumada (2008) considered the model of an ideal observer too, but also considered models of visual acuity for which aberration-free images are used as templates by the observer. Similarly, we introduce a second theoretical observer, which compares the observed data *i*(*x*, *y*) to the unaberrated letters *O*_*k*_(*x*, *y*) by computing the following test statistic:

(4) $$t=\int \;\;\;\int i\left(x,y\right)\left(O_{1,a}\left(x,y\right)-O_{2,a}\left(x,y\right)\right)dxdy$$

*t* is the test statistic of this second observer. We will refer to this theoretical observer as the real observer, which uses as the discriminant *T*(*x*) = *O*_1, *a*_(*x*, *y*) − *O*_2, *a*_(*x*, *y*). The real observer uses unmatched templates to choose between the two classes. We will refer to this observer as real, because using aberration-free images of letters as templates is a plausible model when the experiment is performed with a known alphabet. Unlike the ideal observer, the real observer is not optimal because it does not know the optical aberrations.

Equations 3 and 4 define the test statistics that is computed by each theoretical model. To identify a letter, each model compares its test statistics to a threshold value that we need not to specify in this work, as we seek to compute the theoretical performance of each observer without implementing them with Monte Carlo simulations. In statistical decision theory, *t* and t* are random variables because of noise. The theory quantifies the noise performance of a theoretical observer as the fraction *f* of correct response when the observer performs the binary classification task with fixed model parameters (aberrations, noise level σ, letter size, and contrast). For a binary classification task, the signal to noise ratio (SNR) associated with a test statistic is defined (Barrett & Myers, 2003, p. 819) as the difference between the mean of the test statistic under the hypothesis that the stimulus is of Class 1 or Class 2, divided by the standard deviation of *t* (which is approximately equal for each class). Statistical decision theory relates the SNR to the theoretical fraction *f* of correct classification achieved by the corresponding observer: f=(1+ erf (SNR/2))/2 (Barrett & Myers, 2003, pp. 819–823). For a linear observer, the SNR is computed with standard “error propagation,” noting that *i*(*x*, *y*) is a Gaussian random variable of variance σ^2^ in Equation 1. The SNR of linear observers takes the generic form (Barrett & Myers, 2003, p. 852):

(5) $$\begin{array}{c} \;SNR_{\mathrm{lin}}\left(a\right)=\frac{\left|\int \;\;\;\int T\left(x,y\right)\left(I_{1,a}\left(x,y\right)-I_{2,a}\left(x,y\right)\right)dxdy\right|}{\sigma \sqrt{\int \;\;\;\int T^{2}\left(x,y\right)dxdy}}\;\;\;\;\;\; \end{array}$$

We obtain, using *T*(*x*, *y*) = *I*_1, *a*_(*x*, *y*) − *I*_2, *a*_(*x*, *y*) in Equation 5, for the ideal observer:

(6) $$\;SNR_{t^{*}}\left(a\right)=\frac{1}{\sigma }\sqrt{\int \;\;\;\int {\left(I_{1,a}\left(x,y\right)-I_{2,a}\left(x,y\right)\right)}^{2}dxdy}$$

and we obtain, using *T*(*x*) = *O*_1, *a*_(*x*, *y*) − *O*_2, *a*_(*x*, *y*) in Equation 5, for the real observer :

(7) $$\;SNR_{t}\left(a\right)=\frac{\begin{array}{c} \left|\int \;\;\;\int \left(I_{1,a}\left(x,y\right)-I_{2,a}\left(x,y\right)\right)(O_{1,a}\left(x,y\right)\right.\\ \;-\left.O_{2,a}\left(x,y\right))dxdy\right| \end{array}}{\sigma \sqrt{\int \;\;\;\int {\left(O_{1,a}\left(x,y\right)-O_{2,a}\left(x,y\right)\right)}^{2}dxdy}}$$

For the binary classification, Equation 6 (ideal observer) or Equation 7 (real observer) directly gives the fraction *f* of correct response and can be used to compute the gap (or acuity) threshold if the noise variance σ^2^ is known. In practice, we do not know σ^2^ and only assume that it remains unchanged in all our experiments (for a given subject). For the binary classification problem, we could use Equation 6 or Equation 7 to model the acuity changes due to aberrations of a given subject performing measurements at a fixed fraction *f* of correct response at threshold. To do so, we would compute the letter gap *a* that maintains equal *SNR* when aberrations degrade the visual images of the two classes (*I*_1, *a*_ and *I*_2, *a*_).

### Performance of the ideal observer in a *K*-class problem

$SNR_{t}^{*}$ and *SNR*_*t*_ can be numerically computed for pairs of letters. They both increase when letters are more different, and it is in principle important to take account of all the letters of the alphabet that are used for the visual test. To do so, statistical decision theory introduces data separability (Barrett & Myers, 2003, p. 852), which quantifies the optimal SNR achievable by the ideal observer in a so-called K-class problem (a visual acuity test with K letters). Dalimier and Dainty (2008) built their model of contrast sensitivity measurements using data separability. Data separability S*(*a*) is a metric of performance for the ideal observer and can be written as

(8) $$\begin{array}{c} \;S^{*}\left(a\right)=\frac{2}{\sigma \sqrt{K}}\sqrt{\sum_{k=1}^{K}\int \;\;\;\int {\left(I_{k,a}\left(x,y\right)-{\bar{I}}_{a}\left(x,y\right)\right)}^{2}dxdy}\;\;\;\; \end{array}$$

where ${\bar{I}}_{a}\left(x,y\right)=\sum_{k=1}^{K}I_{k,a}\left(x,y\right)$. As Dalimier and Dainty, we have scaled the definition of S* in order to have $SNR_{t^{*}}\left(a\right)=S^{*}\left(a\right)$ for *K* = 2. We note that S* corresponds to the root mean square value of *K* values of $SNR_{t^{*}}$, which correspond to *K* hypothetical binary classification problems of choosing between *I*_*k*, *a*_ and ${\bar{I}}_{a}$. Data separability quantifies the overall difference between the visual images of each letter used for the test, as a single number that is normalized by the level of noise σ.

### Performance of the real observer in a *K*-class problem

By analogy, we formulate the *S*(*a*) metric of performance for the real observer in the K-class problem. We compute the root mean square value of *K* values of *SNR*_*t*_ (Equation 7) that correspond to hypothetical binary classification problems of choosing between *I*_*k*, *a*_ and ${\bar{I}}_{a}$:

(9) $$\begin{array}{c} \;S\left(a\right)=\frac{2}{\sigma \sqrt{K}}\sqrt{\sum_{k=1}^{K}\frac{\begin{array}{c} (\int \;\;\;\int \left(I_{k,a}\left(x,y\right)-{\bar{I}}_{a}\left(x,y\right)\right)\\ \;\;\;\;\times \left(O_{k,a}\left(x,y\right)-{\bar{O}}_{a}\left(x,y\right)\right)dxdy{)}^{2} \end{array}}{\int \;\;\;\int {\left(O_{k,a}\left(x,y\right)-{\bar{O}}_{a}\left(x,y\right)\right)}^{2}dxdy}}\;\;\; \end{array}$$

We also have *S*(*a*) = *SNR*_*t*_(*a*) for *K* = 2, and we will refer to *S*(*a*) as the data separability of the real observer.

As long as gap threshold corresponds to a fixed percentage of correct response (Dalimier & Dainty, 2008), two measurements of visual acuity (with and without aberrations, corresponding to the inverse of letter gap threshold *a*_*B*_ and *a*_0_, respectively) are related by equal data separability ($S_{0}^{*}\left(a_{0}\right)=S_{B}^{*}\left(a_{B}\right)$ or *S*_0_(*a*_0_) = *S*_*B*_(*a*_*B*_), depending on which model of observer we rely on). The 0 index corresponds to the reference (aberration-free) condition, and the *B* index corresponds to the condition with aberration. Dalimier and Dainty (2008) noted that data separability is proportional to stimulus contrast and predicted the ratio (with/without aberration) of contrast sensitivity measurements as the ratio of data separability with unitary stimulus contrast. Our goal is to use data separability to predict the ratio of letter gap threshold, which is the inverse of decimal visual acuity. Because S* and *S* are not proportional to letter gap *a*, we introduce below the numerical method that we implemented for each of the two observers.

### Model of visual images

In this work, we model visual images using standard Fourier optics calculations. The visual images take the form of two-dimensional functions of spatial coordinates, and it is important to emphasize at this point that other approaches exist. One can, for instance, take account of the finite bandwidth of independent visual channels (Sachs, Nachmias & Robson, 1971) that are tuned to the spatial spectra of letters (Majaj, Pelli, Kurshan, & Palomares, 2002), and with this approach, model equations take a more algebraic form (Myers & Barrett, 1987; Dalimier, 2007). Here, the visual image that corresponds to the k th  letter choice can be written as

(10) $$\begin{array}{ccc} I_{k,a}\left(x,y\right) & \;= & F^{-1}\left\{NTF\left(f_{x},f_{y}\right)OTF\left(f_{x},f_{y}\right)\right.\\ & & \times \left.F\left\{O_{k,a}\left(x,y\right)\right\}\right\} \end{array}$$

*O*_*k*, *a*_(*x*, *y*) models the luminance distribution of stimulus for the k th  letter choice and the *a* letter gap. *OTF* is the optical transfer function, which we will define in Equation 10 as either *OTF*_0_ (condition 0, without aberration) or *OTF*_*B*_ (condition *B*, with aberration). *NTF* is the neural transfer function of the eye, which we defined using a generic model that combines different studies from the literature (Hastings, Marsack, Thibos, & Applegate, 2020). F denotes the Fourier transform operator. We considered monochromatic (λ = 530 nm) and monocular vision in this work.

### Numerical method to predict letters lost for theoretical observers

Following pioneering studies of the effect of aberrations on visual acuity (Applegate, Marsack, Ramos, & Sarver, 2003; Applegate, Ballentine, Gross, Sarver, & Sarver, 2003), we aimed at predicting the number of letters lost, due to aberration, on the logMAR chart. As one line of the logMAR chart has five letters and corresponds to a 0.1 variation of the logarithm of letter gap, the number of letters lost equals −50 times the difference (with/without aberration) in logMAR visual acuity. The model of an ideal observer predicts a number L* of letters lost as

(11) $$L^{*}=-50\log_{10}\left(\frac{a_{B}^{*}}{a_{0}}\right)\;\text{with}\;S_{B}^{*}\left(a_{B}^{*}\right)=S_{0}^{*}\left(a_{0}\right)$$

and the model of a real observer predicts a number *L* of letters lost as

(12) $$L=-50\log_{10}\left(\frac{a_{B}}{a_{0}}\right)\;\text{with}\;S_{B}\left(a_{B}\right)=S_{0}\left(a_{0}\right)$$

We compute numerically data separability as a function of letter gap *a* by combining Equations 8 and 10 for the ideal observer, as well as Equations 9 and 10 for the real observer. This calculation is performed without aberration (functions $S_{0}^{*}\left(a\right)$ and *S*_0_(*a*) for the ideal and real observers, respectively) and with aberration (functions $S_{B}^{*}\left(a\right)$ and *S*_*B*_(*a*) for the ideal and real observers, respectively). Our numerical method requires to first arbitrarily set the gap threshold, *a*_0_, in the aberration-free condition. We set *a*_0_ = 1 arcminute for both observers, and we numerically find the letter gap $a_{B}^{*}$ for which $S_{B}^{*}\left(a_{B}^{*}\right)=S_{0}^{*}\left(a_{0}\right)$ (ideal observer) and *a*_*B*_ for which *S*_*B*_(*a*_*B*_) = *S*_0_(*a*_0_) (real observer). To solve these two equations, we use linear fits of the $S_{B}^{*}$, $S_{0}^{*}$, *S*_*B*_, and *S*_0_ functions on a logarithmic scale.

**Figure 1.**
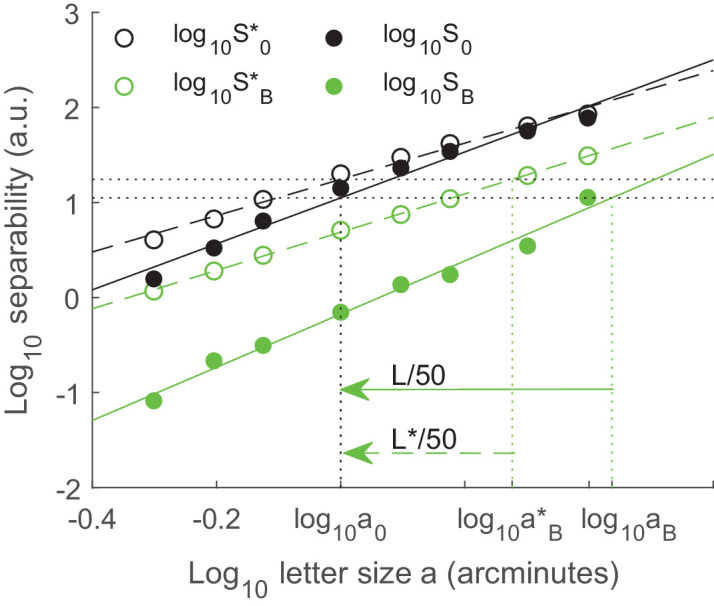
Logarithm of data separability of the ideal observer as a function of logarithm of letter gap *a*, with aberration ($S_{B}^{*}$, open green circle) and without aberration ($S_{0}^{*}$, open black circle). The linear fits of $S_{B}^{*}$ and $S_{0}^{*}$ (dashed green and dashed black lines, respectively), on the logarithmic scale, allow us to find $a_{B}^{*}$ such that $S_{B}^{*}\left(a_{B}^{*}\right)=S_{0}^{*}\left(a_{0}\right)$. We arbitrarily set log_10_*a*_0_ = 0. The same approach is implemented for the real observer (*S*_0_: filled black circle and *S*_*B*_: filled green circle), in order to solve *S*_*B*_(*a*_*B*_) = *S*_0_(*a*_0_). For the model of the ideal observer, letters lost L* = −13.8 (= 50 × the amplitude of the dashed arrow). For the model of the real observer, letters lost *L* = −21.9 (= 50 × the amplitude of the solid arrow).

Figure 1 shows our numerical method in detail. Condition *B* here corresponds to +0.55 diopters of defocus for a 5-mm pupil diameter, and condition 0 corresponds to a diffraction-limited eye of pupil diameter 5 mm. We set *a*_0_ = 1 arcminute, and compute $\log_{10}S_{0}^{*}\left(a_{0}\right)=1.24$ using the linear fit of $\log_{10}S_{0}^{*}$ (dashed black line). We use the linear fit of $\log_{10}S_{B}^{*}$ (dashed green line) to solve $\log_{10}S_{B}^{*}\left(a_{B}^{*}\right)=1.24$ and obtain $a_{B}^{*}=1.89$ arcminutes, which corresponds to L* = −50 × (log_10_1.89) = −13.8 letters for the ideal observer. We use the same method for the real observer (linear fits on the logarithmic scale are the solid black line (*S*_0_) and the solid green line (*S*_*B*_)), and obtain *L* = −21.9 letters. For each observer, the linear fits are approximately parallel on the logarithmic scale, so that the predicted letters lost barely depends on the arbitrary reference value of *a*_0_ = 1 arcminute.

### Approximations of letters lost, using model-based metrics

Computing data separability for different letter gaps is relatively computationally expensive. It may not be practical for clinical applications of aberrometry, as it requires numerical computations of visual images that are cumbersome. Here we find the metric of visual image quality that approximates the letters lost for each theoretical model of observer.

#### Metric based on the model of an ideal observer

We first detail the computational aspect of S* using Fourier optics formalism. We introduce ${\tilde{\Delta }}_{k,a}\left(f_{x},f_{y}\right)$, which is the Fourier transform of the difference between the stimulus letter *O*_*k*, *a*_ and the average (across letters) ${\bar{O}}_{a}$:

(13) $${\tilde{\Delta }}_{k,a}\left(f_{x},f_{y}\right)=F\left\{O_{k,a}\left(x,y\right)-{\bar{O}}_{a}\left(x,y\right)\right\}$$

S* can be written as

(14) $$\begin{array}{c} \;S^{*}\left(a\right)=\frac{2}{\sigma \sqrt{K}}\sqrt{\begin{array}{c} \sum_{k=1}^{K}\int \;\;\;\int |F^{-1}\left\{NTF(f_{x},f_{y})OTF\right.\\ \;\left.\left(f_{x},f_{y}\right){\tilde{\Delta }}_{k,a}\left(f_{x},f_{y}\right)\right\}{|}^{2}dxdy \end{array}}\;\; \end{array}$$

Using Parseval’s identity, we obtain the following formulation of S*:

(15) $$\begin{array}{c} \;S^{*}\left(a\right)=\frac{2}{\sigma \sqrt{K}}\sqrt{\begin{array}{c} \sum_{k=1}^{K}\int \;\;\;\int |NTF\left(f_{x},f_{y}\right)OTF\\ \;\left(f_{x},f_{y}\right){\tilde{\Delta }}_{k,a}\left(f_{x},f_{y}\right){|}^{2}df_{x}df_{y} \end{array}}\; \end{array}$$

The scaling property of Fourier transforms gives

(16) $${\tilde{\Delta }}_{k,a}\left(f_{x},f_{y}\right)=a^{2}{\tilde{\Delta }}_{k,1}\left(af_{x},af_{y}\right)$$

In the limit of small Sloan letters, the ${\tilde{\Delta }}_{k,1}\left(af_{x},af_{y}\right)$ spectrum can be approximated by a constant function ${\tilde{\Delta }}_{k}$ that extends over the full domain of spatial frequency (and therefore does not depend either on (*f*_*x*_, *f*_*y*_) or on *a*). We had used this approximation to define a model-based metric, *M*, which predicts contrast sensitivity changes with optical aberrations (Leroux et al., 2022). We obtain the approximated form of S*, which is a quadratic function of letter gap *a*:

(17) $$\begin{array}{ccc} & & \;S^{*}\left(a\right)\approx a^{2}\frac{2}{\sigma \sqrt{K}}\sqrt{\sum_{k=1}^{K}{\left|{\tilde{\Delta }}_{k}\right|}^{2}}\\ & & \;\times \;\sqrt{\int \;\;\;\int {\left(NTF\left(f_{x},f_{y}\right)MTF\left(f_{x},f_{y}\right)\right)}^{2}df_{x}df_{y}}\; \end{array}$$

Using this approximation for $S_{0}^{*}\left(a_{0}\right)$ and $S_{B}^{*}\left(a_{B}^{*}\right)$, the $S_{0}^{*}\left(a_{0}\right)=S_{B}^{*}\left(a_{B}^{*}\right)$ equality of Equation 11 can be written as

(18) $$\begin{array}{ccc} & & \;a_{0}^{2}\sqrt{\int \;\;\;\int {\left(NTF\left(f_{x},f_{y}\right)MTF_{0}\left(f_{x},f_{y}\right)\right)}^{2}df_{x}df_{y}}\\ & & \;\approx a_{B}^{*2}\sqrt{\int \;\;\;\int {\left(NTF\left(f_{x},f_{y}\right)MTF_{B}\left(f_{x},f_{y}\right)\right)}^{2}df_{x}df_{y}}\; \end{array}$$

The letters lost $L^{*}=-50\log_{10}\left(a_{B}^{*}/a_{0}\right)$ (Equation 11) can therefore be approximated as

(19) $$\begin{array}{ccc} & & \;L^{*}\approx \frac{50}{4}\log_{10}\\ & & \;\left(\frac{\int \;\;\;\int {\left(NTF\left(f_{x},f_{y}\right)MTF_{B}\left(f_{x},f_{y}\right)\right)}^{2}df_{x}df_{y}}{\int \;\;\;\int {\left(NTF\left(f_{x},f_{y}\right)MTF_{0}\left(f_{x},f_{y}\right)\right)}^{2}df_{x}df_{y}}\right)\; \end{array}$$

The argument of the logarithm in Equation 19 is the square of the *M* metric, which we defined to predict ratios (with/without aberration) of contrast sensitivity measurements from the model of the ideal observer (Leroux et al., 2022; Leroux et al., 2023):

(20) $$\begin{array}{c} \;M={\left(\frac{\int \;\;\;\int {\left(NTF\left(f_{x},f_{y}\right)MTF_{B}\left(f_{x},f_{y}\right)\right)}^{2}df_{x}df_{y}}{\int \;\;\;\int {\left(NTF\left(f_{x},f_{y}\right)MTF_{0}\left(f_{x},f_{y}\right)\right)}^{2}df_{x}df_{y}}\right)}^{1/2}\; \end{array}$$

Hence, we obtain the approximation of letters lost for the ideal observer as a metric of visual image quality:

(21) $$L^{*}\approx 25\log_{10}\left(M\right)$$

*M* is comparable to the VSMTF metric (Thibos et al., 2004), in the sense that it is computed as an integral that combines the MTF and the NTF. However, the power of 2 in Equation 20 is specific to *M* and does not appear in the definition of VSMTF. The consequence of this power is to give more weight to the spatial frequencies for which the MTF is high (i.e., the lower spatial frequencies).

#### Metric based on the model of the real observer

For the model of a real observer, we insert ${\tilde{\Delta }}_{k,a}\left(f_{x},f_{y}\right)$ in Equation 9 to write data separability *S* as

(22) $$\begin{array}{c} \;S\left(a\right)=\frac{2}{\sigma \sqrt{K}}\sqrt{\sum_{k=1}^{K}\frac{\begin{array}{c} (\int \;\;\;\int F^{-1}\left\{NTF(f_{x},f_{y})OTF\right.\\ \;\;\;\;\left.\left(f_{x},f_{y}\right){\tilde{\Delta }}_{k,a}\left(f_{x},f_{y}\right)\right\}\\ \;\;\;\;F^{-1}\left\{{\tilde{\Delta }}_{k,a}\left(f_{x},f_{y}\right)\right\}dxdy{)}^{2} \end{array}}{\int \;\;\;\int {\left(F^{-1}\left\{{\tilde{\Delta }}_{k,a}\left(f_{x},f_{y}\right)\right\}\right)}^{2}dxdy}}\; \end{array}$$

We use Parseval’s theorem for both the numerator and denominator in Equation 22. Noting that $F^{-1}\left\{{\tilde{\Delta }}_{k,a}\left(f_{x},f_{y}\right)\right\}=O_{k,a}\left(x,y\right)-{\bar{O}}_{a}\left(x,y\right)$ is real-valued, we obtain

(23) $$\begin{array}{c} \;S\left(a\right)=\frac{2}{\sigma \sqrt{K}}\sqrt{\sum_{k=1}^{K}\frac{\begin{array}{c} (\int \;\;\;\int NTF\left(f_{x},f_{y}\right)OTF\left(f_{x},f_{y}\right)\\ \;\;\;\;{\left|{\tilde{\Delta }}_{k,a}\left(f_{x},f_{y}\right)\right|}^{2}df_{x}df_{y}{)}^{2} \end{array}}{\int \;\;\;\int {\left|{\tilde{\Delta }}_{k,a}\left(f_{x},f_{y}\right)\right|}^{2}df_{x}df_{y}}}\; \end{array}$$

For the numerator of Equation 23, we use the small letter approximation that we used to approximate the ideal observer with the 25log_10_(*M*) metric (Equations 15 to 17). We obtain

(24) $$\begin{array}{ccc} & & \;S\left(a\right)\approx a^{4}\frac{2}{\sigma \sqrt{K}}\sqrt{\sum_{k=1}^{K}\frac{|{\tilde{\Delta }}_{k}{|}^{4}}{\int \;\;\;\int {\left|{\tilde{\Delta }}_{k,a}\left(f_{x},f_{y}\right)\right|}^{2}df_{x}df_{y}}}\\ & & \;\times \left|\int \;\;\;\int NTF\left(f_{x},f_{y}\right)OTF\left(f_{x},f_{y}\right)df_{x}df_{y}\right|\; \end{array}$$

The denominator in Equation 24 still depends on *a*, so it needs to be rearranged. It is not well approximated by the small letter approximation, which would here diverge because there is no weighting function (other than ${\left|{\tilde{\Delta }}_{k,a}\left(f_{x},f_{y}\right)\right|}^{2}$) in the integral (over $R^{2}$). We make use of Equation 16, and with a change of variable. we find

$\int \;\;\;\int {\left|{\tilde{\Delta }}_{k,a}\left(f_{x},f_{y}\right)\right|}^{2}df_{x}df_{y}=a^{2}\int \;\;\;\int {\left|{\tilde{\Delta }}_{k,1}\left(f_{x},f_{y}\right)\right|}^{2}df_{x}df_{y}$ .

We obtain the approximated form of *S*(*a*), which is a cubic function of letter gap *a*:

(25) $$\begin{array}{ccc} & & \;S\left(a\right)\approx a^{3}\frac{2}{\sigma \sqrt{K}}\sqrt{\sum_{k=1}^{K}\frac{|{\tilde{\Delta }}_{k}{|}^{4}}{\int \;\;\;\int {\left|{\tilde{\Delta }}_{k,1}\left(f_{x},f_{y}\right)\right|}^{2}df_{x}df_{y}}}\\ & & \;\times \left|\int \;\;\;\int NTF\left(f_{x},f_{y}\right)OTF\left(f_{x},f_{y}\right)df_{x}df_{y}\right|\; \end{array}$$

Using this approximation for *S*_0_(*a*_0_) and *S*_*B*_(*a*_*B*_), the *S*_0_(*a*_0_) = *S*_*B*_(*a*_*B*_) equality (Equation 12) can be written as:

(26) $$\begin{array}{ccc} & & \;a_{0}^{3}\left|\int \;\;\;\int NTF\left(f_{x},f_{y}\right)OTF_{0}\left(f_{x},f_{y}\right)df_{x}df_{y}\right|\approx \\ & & \;\;a_{B}^{3}\left|\int \;\;\;\int NTF\left(f_{x},f_{y}\right)OTF_{B}\left(f_{x},f_{y}\right)df_{x}df_{y}\right|\; \end{array}$$

The letters lost *L* = −50log_10_(*a*_*B*_/*a*_0_) (Equation 12) can therefore be approximated as

(27) $$\begin{array}{c} \;L\approx \frac{50}{3}\log_{10}\left(\frac{\left|\int \;\;\;\int NTF\left(f_{x},f_{y}\right)OTF_{0}\left(f_{x},f_{y}\right)df_{x}df_{y}\right|}{\left|\int \;\;\;\int NTF\left(f_{x},f_{y}\right)OTF_{B}\left(f_{x},f_{y}\right)df_{x}df_{y}\right|}\right)\;\;\;\; \end{array}$$

The argument of the logarithm in Equation 27 is the modulus of the visual Strehl computed with the optical transfer function (*VSOTF*) metric, as primarily introduced by Thibos et al. (2004):

(28) $$\;VSOTF=\frac{\int \;\;\;\int NTF\left(f_{x},f_{y}\right)OTF_{0}\left(f_{x},f_{y}\right)df_{x}df_{y}}{\int \;\;\;\int NTF\left(f_{x},f_{y}\right)OTF_{B}\left(f_{x},f_{y}\right)df_{x}df_{y}}$$

Hence, we obtain the approximation of letters lost for the real observer as a metric of visual image quality:

(29) $$L\approx \frac{50}{3}\log_{10}\left|VSOTF\right|$$

#### Numerical examples

**Figure 2.**
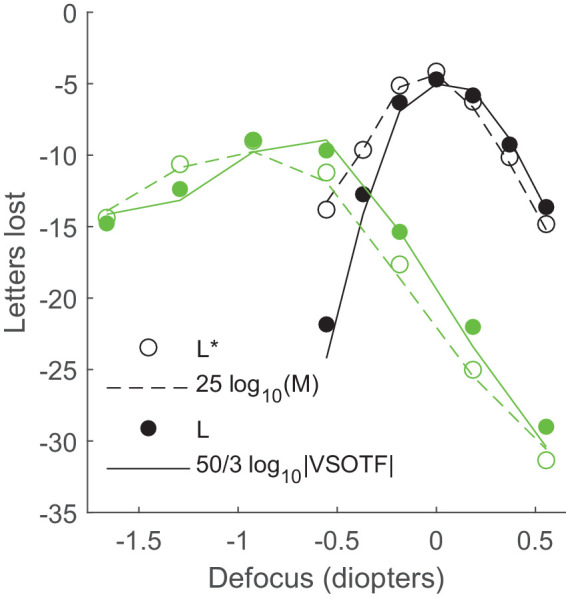
Through-focus calculations of letters lost, for two fixed amplitudes of spherical aberration. Black curves: $z_{4}^{0}=0.1$ μm. Green curves: $z_{4}^{0}=0.2$ μm. L* (open circles) and *L* (filled circles) are the predictions of the complete model of the ideal and real observers, respectively. The corresponding approximations, as metrics of visual image quality, are 25log_10_(*M*) (dashed lines) and 50/3log_10_|*VSOTF*| (solid lines), respectively.

We show in Figure 2 the accuracy of the approximating model of letters lost with a metric of visual image quality, for the ideal observer (Equation 21: open circle for L* and dashed line for 25log_10_(*M*)) and for the real observer (Equation 29: filled circle for *L* and solid line for 50/3log_10_|*VSOTF*|). Black and green curves correspond to through-focus calculations with an additional fixed amount of Zernike spherical aberration $z_{4}^{0}=0.1$ μm and $z_{4}^{0}=0.2$ μm, respectively. We have used a 5-mm pupil size for the conditions with aberration (index *B* in the model equations) and without aberration (index 0 in the model equations). The overall root mean square error between the complete model and its metric is 0.52 letters for the ideal observer (25log_10_(*M*) − L*) and 1.02 letters for the real observer (50/3log_10_|*VSOTF*| − *L*).

## Methods

### Stimulus display

We have used the computational approach to measure the effect of optical aberrations on visual acuity (Burton & Haig, 1984; Applegate, Marsack et al., 2003). The displayed letters were convolved with a numerical point spread function, which we defined with Zernike aberrations for a 5-mm pupil diameter at the 530-nm wavelength. Four experiments consisted of varying $z_{2}^{0}$ Zernike defocus, with an additional fixed amount of Zernike spherical aberration ($z_{4}^{0}=0,0.1,0.2,0.3$ μm). A fifth experiment consisted of varying $z_{3}^{-1}$ Zernike coma alone. Each experiment consisted of seven charts with varying amplitude of defocus or coma, plus one control chart (without aberration). The eight charts appeared in a randomized order to limit the effect of blur adaptation on the measurements (Artal et al., 2004; Sawides et al., 2010; de Gracia, Dorronsoro, Marin, Hernandez, & Marcos, 2011; Ohlendorf, Tabernero, & Schaeffel, 2011; Sawides, de Gracia, Dorronsoro, Webster, & Marcos, 2011). Each subject only performed two experiments (2 × 8 charts) in order to limit learning and fatigue effects. We have used a custom-made program using MATLAB functions from the Psychophysics Toolbox (Brainard, 1997).

We have used the standard set of letters for testing visual acuity in the United States (D, H, N, V, R, Z, S, K, O, C) (Sloan, 1959; Pelli, Robson, & Wilkins, 1988; Ricci, Cedrone, & Cerulli, 1998). We have used black Sloan letters on a green background, with the maximum contrast permitted by the 8-bit green channel. The spectrum of the green channel was measured with a spectrometer (HR2000+, Ocean Optics) as Gaussian shaped (center at 530 nm, 43 nm full width at half maximum) and was estimated to be sufficiently narrow to neglect chromatic aberrations. Subjects performed the experiments with ambient light, which we measured with a calibrated luxmeter (RS Pro TES-1332- G, RS components). This light corresponded to 337 Td retinal illuminance, for the average 3.1-mm pupil of the experiment. We also calculated the retinal illuminance of the test chart alone, which was 706 Td for the average 3.1-mm pupil diameter.

### Subjects

Twenty informed, yet untrained, subjects took part in the study. Subjects wore their current refractive correction and monocularly looked at the test screen (Dell Ultrasharp U2720Q) with their dominant eye. We used the Porta test of sighting dominance. We measured the pupil diameter of each subject in the condition of the test with a ruler. The average age was 28 years (± 7 years standard deviation), the average pupil was 3.1 mm diameter (± 0.7 mm standard deviation). The average spherical equivalent correction was −0.14 diopters (± 1.1 diopters standard deviation), and the average cylindrical correction was −0.11 diopters (± 0.25 diopters standard deviation). Prior informed consent was obtained from the subjects. This study was reviewed by an independent ethical review board and conforms with the principles and applicable guidelines for the protection of human subjects in biomedical research. The experiment was performed according to the Declaration of Helsinki on human experimentation.

### Measurements of letters lost

To measure letters lost, we used the same termination rule as Applegate, Balentine et al. (2003): We counted letters read on a logMAR chart until five errors occurred cumulatively in the chart. Because of the size of the screen, we only displayed the last nine lines of the logMAR chart, which corresponded to visual acuity ranging from 20/63 to 20/10.

### Measurements of visual acuity in the control condition

We measured visual acuity with the aberration-free chart. We assigned a score of 0.02 logMAR for each letter read until five errors occurred cumulatively in the chart. Because our chart started at the 20/63 line (0.5 logMAR), the visual acuity was estimated as 0.6 − 0.02 × *n*_0_ logMAR when *n*_0_ letters were read. For each subject, we reported the average of two measurements.

### Data analysis

For each aberration level of each experiment, we averaged the number of letters lost across eight different subjects. As mentioned above, each subject completed only two experiments so we did not have all 20 subjects per experiment. The predictive performance of models and metrics was evaluated with respect to the intersubject averaged measurements to reduce the effect of measurement noise, which may have been high because subjects were not trained to the task of the experiment. Moreover, models and metrics were not customized to the subject’s visual system and did not aim at describing intersubject differences. We quantified the performance of models and metrics by computing the root mean square value ϵ of the (average measurement model) difference and we also computed the (α, β) parameters of the (averagemeasurement=α× model +β) linear fit.

### Model predictions

We computed the 25log_10_(*M*) and 50/3log_10_|*VSOTF*| metrics using Equation 20 and Equation 28, respectively. For both metrics, we defined *OTF*_*B*_ as the product of the transfer function that we used to numerically blur the displayed Sloan letters for the experiment, times another transfer function that modeled the process of viewing the Sloan letter with a supposedly diffraction-limited eye of pupil diameter 3.1 mm (the mean pupil size). This latter transfer function alone also defined *OTF*_0_ in the denominator of Equation 20 and Equation 28. The *NTF* was computed with the code given by Hastings et al. (2020), for the mean age of subjects (28 years) and the retinal illuminance of our experiment (706 Td). In this study, the model does not take account of the subject’s optical aberrations, as the *B* condition only refers to the numerical blur. This computation of the two metrics approximately matches our experiment that combines numerical blur (over a 5-mm pupil) and optical blur (over a 3.1-mm pupil on average), assuming that the subject’s optics are diffraction-limited for a 3.1-mm pupil with their current refraction. While this assumption is certainly optimistic (Hastings et al., 2018), we rely on the relative nature of the letters lost measurements to reduce the impact of the eye’s wavefront errors after correction (Applegate, Marsack et al., 2003). The computation of the L* (Equation 11 and Equation 15) and *L* (Equation 12 and Equation 23) models of letters lost were performed using the same transfer functions.

## Results

In the control condition (aberration free), the visual acuity was −0.21 ± 0.05 logMAR (mean ± standard deviation). All subjects had better than 20/20 visual acuity. The logMAR values were in the (− 0.27, −0.12) range.

**Figure 3.**
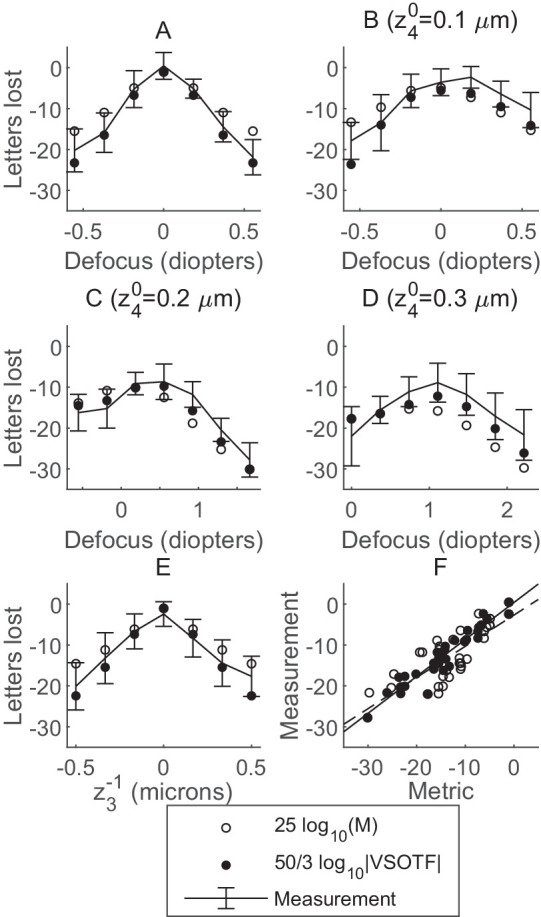
Comparison of the two model-based metrics with experimental measurements of letters lost. Error bars show the average ± standard deviation (across eight subjects) of the measured letters lost as a function of varying defocus $z_{2}^{0}$ and fixed spherical aberration: $z_{4}^{0}=0$ (A), $z_{4}^{0}=0.1\;\mu$m (B), $z_{4}^{0}=0.2\;\mu$m (C), $z_{4}^{0}=0.3\;\mu$m (D), and as a function of varying coma $z_{3}^{-1}$ alone (E). The two model-based metrics are shown for each condition: 25log_10_(*M*) (open circle) and 50/3log_10_|*VSOTF*| (filled circle). (F) The corresponding scatter graphs of all (average measurement, metric) pairs. Dashed and solid lines show the corresponding linear fits for 25log_10_(*M*) and 50/3log_10_|*VSOTF*|, respectively. Fit parameters are given in Table 1.

Figure 3 compares the two metrics (25log_10_(*M*): open circle; 50/3log_10_|*VSOTF*|: filled circle) to experimental measurements of letters lost. We show as error bars in Figures 3A–E the average ± standard deviation (across eight subjects) of the measured letters lost, as a function of the varying amplitude of aberration. Figures 3A–D corresponds to the experiments with varying Zernike defocus $z_{2}^{0}$ and fixed spherical aberration: $z_{4}^{0}=0$ (A), $z_{4}^{0}=0.1\;\mu$m (B), $z_{4}^{0}=0.2\;\mu$m (C), and $z_{4}^{0}=0.3\;\mu$m (D). Figure 3E corresponds to the experiment with varying coma $z_{3}^{-1}$. The corresponding scatter graphs of all (average measurement, metric) pairs are shown in Figure 3F. The dashed and solid lines show the corresponding linear fits for 25log_10_(*M*) and 50/3log_10_|*VSOTF*|, respectively.

The parameters of the linear fit (averagemeasurement=α× model +β) are given in Table 1, for each model and metric. The highest coefficient of determination is *r*^2^ = 0.92 for the 50/3log_10_|*VSOTF*| metric. The second highest coefficient of determination is *r*^2^ = 0.91 for the *L* model. The best agreement of fit parameters with the *y* = *x* perfect agreement line is for the *L* model (α = 0.94, close to unity, and β = 0.24 letters read). The second best agreement of fit parameters with the *y* = *x* perfect agreement line is for the 50/3log_10_|*VSOTF*| metric (α = 0.91 and β = 0.38 letters read).

**Table 1.** Comparison of models to measurements. ϵ is the root mean square value of the (average measurement model) difference, for each model (L*, *L*, 25log_10_(*M*), 50/3log_10_|*VSOTF*|). We give the coefficients of the (averagemeasurement=α× model +β) linear fit. *r*^2^ is the coefficient of determination of the fit, which is shown in Figure 3F for the 25log_10_(*M*) metric and the 50/3log_10_|*VSOTF*| metric.

	ϵ	α	β	*r* ^2^
L*	3.95	0.79	−2.46	0.69
*L*	2.26	0.94	0.24	0.91
25log_10_(*M*)	4.32	0.76	−2.69	0.64
50/3log_10_|*VSOTF*|	2.71	0.91	0.38	0.92

The overall root mean square value ϵ of the (average measurement model) difference is given in Table 1 for each model. The lowest value is ϵ = 2.26 letters for the *L* model. The second lowest value is ϵ = 2.71 letters for the corresponding model-based metric, 50/3log_10_|*VSOTF*|.

## Discussion

The main contribution of this work is to relate two existing metrics of visual image quality to the underlying theoretical models of linear observers. The two metrics predict the number of letters lost (negative number) on the logMAR chart, due to optical aberrations. The benefit of our theoretical approach is twofold. First, we aim at predicting letters lost (or acuity changes) without needing a posteriori scale or offset. Second, it gives the interpretation of each metric as a subject’s strategy to identify optotypes.

### Predicting letters lost (or acuity changes) without needing a posteriori scale or offset

In agreement with studies that correlate the *VSOTF* metric with visual acuity measurements (Marsack et al., 2004; Cheng et al., 2004; Ravikumar et al., 2013), we find that the 50/3log_10_|*VSOTF*| metric has a high coefficient of determination (*r*^2^ = 0.92 in Table 1). Our prediction of acuity changes makes it possible to go beyond mere analysis of the *r*^2^ coefficient, by comparing the parameters of the (measurement, model) linear fit with the *y* = *x* perfect agreement line. We find that the 50/3log_10_|*VSOTF*| metric has a slope near unity (α = 0.91) and intercept near zero (β = 0.38 letters; see Table 1). Metrics of letters lost can be converted to predict acuity change after multiplication by a factor of −1/50 (one lost line is −5 letters lost, or +0.1 logMAR). Hence, we predict logMAR acuity changes as −1/3log_10_|*VSOTF*|. This prediction approximately agrees with the (logMAR acuity changes, log_10_|*VSOTF*|) scatter graph reported by (Ravikumar et al., 2013, Figure 5) for a set of normal wavefront errors: Their linear fit is $\mathrm{logMAR}=-0.190\log_{10}\left|VSOTF\right|+0.0420$. When comparing this linear fit to our theoretical prediction (−1/3log_10_|*VSOTF*|), the mean absolute value of the difference in predicted visual acuity is 0.083 logMAR for the aberrations studied in our work.

### Interpretation of each metric as a subject’s strategy to identify optotypes

In this work, the prediction with the 25log_10_(*M*) metric is poorer than with the 50/3log_10_|*VSOTF*| metric, both in terms of coefficient of determination (*r*^2^ = 0.64 vs *r*^2^ = 0.92) and parameters of the linear fit that differ from the *y* = *x* perfect agreement (α = 0.76 and β = −2.69 letters vs. α = 0.91 and β = 0.38 letters in Table 1). We hypothesize that the better prediction with the 50/3log_10_|*VSOTF*| metric originates from a more suitable model of a theoretical observer. As also shown in Table 1, the model of a real observer (*L*) better agrees with measurements than the ideal observer (L*). We recall that the real observer essentially projects visual images on a set of unaberrated letters, while the ideal observer uses aberrated letters as templates. Using aberrated images is an optimal strategy because they properly represent the observed visual images. Indeed, the ideal observer sets the upper bound of visual acuity for given experimental conditions (aberration, noise level, letter contrast). The model of the ideal observer predicts the optimal visual acuity, both with and without aberrations. Counterintuitively, this model can predict higher acuity loss with optical aberrations than the real observer. It is so in 40% of the aberration conditions analyzed in Figure 3. Similarity, the −25log_10_(*M*) metric, which is based on the model of an ideal observer, can predict more letters lost than the −50/3log_10_|*VSOTF*| metric. Watson and Ahumada (2008) used Monte Carlo simulations of acuity testing to compare the data agreement of two correlation-maximizing observers: the observer that uses unaberrated letters as a set of templates (XL observer in their Table 2) and the observer that uses aberrated letters (XA in their Table 2). They obtained a better prediction of absolute visual acuity (lower root mean square error) with aberrated templates when the noise level of the model maximized data agreement, but their results also show that unaberrated templates can give better prediction for other levels of noise (see their Figure 6). In that situation, the Watson and Ahumada model agrees with our results, as we obtain better prediction with unaberrated templates (*L* and 50/3log_10_|*VSOTF*|) than with aberrated templates (L* and 25log_10_(*M*)). The predicted acuity changes do not depend on the noise level σ, which cancels out when writing that data separability at threshold remains unchanged when aberrations change (Equation 18 and 26 for the ideal and real observers, respectively). Like Watson and Ahumada, we quantify the agreement between a model and measurements with the root mean square value of the (measurement model) difference (ϵ, see Table 1). With ϵ = 2.71 letters for the 50/3log_10_|*VSOTF*| metric, the root mean square difference corresponds to around 0.05 logMAR acuity, which is similar to the errors given by Watson and Ahumada (2008) in their Figure 6.

### Role of the phase transfer function

In this study, better prediction of acuity measurements with the 50/3log_10_|*VSOTF*| metric than with the 25log_10_(*M*) metric corroborates experimental evidence that the phase transfer function of the eye impacts visual acuity measurements (Piotrowski & Campbell, 1982; Sarver & Applegate, 2004; Ravikumar, Bradley, & Thibos, 2010), as *VSOTF* depends on the *OTF* while *M* only depends on its modulus (the *MTF*).

### Comparison with contrast sensitivity

In our previous study (Leroux et al., 2023), we reported on the prediction of contrast sensitivity measurements with similar metrics and found different results: higher *r*^2^, and better agreement with the *y* = *x* line, for *M* than for *VSOTF*. This result favored the model of an ideal observer for contrast sensitivity measurements, unlike the present study of visual acuity. We think that this difference can be explained by the specific effect of aberrations during each visual test. During a contrast sensitivity measurement, optical blur remains the same for optotypes of fixed size and varying contrast. Hence, the model of an ideal observer only requires one set of aberrated letters to classify letters that all have the same size. During a visual acuity measurement, the model of an ideal observer requires size-dependent sets of aberrated templates. Moreover, the effect of optical blur is exacerbated for small letters. Most subjects probably lose track during this “heavy computational task,” and their visual performance is not well modeled by an ideal observer. The discrepancy of human subjects with the ideal observer is probably specific to our study on the effect of aberrations on visual acuity. The comparison of a subject’s visual performance with the performance of the ideal observer is usually represented as a ratio named *efficiency* (Pelli, Burns, Farell, & Moore-Page, 2006; Watson & Ahumada, 2012, Watson & Ahumada, 2015). In vision science (Geisler, 2011) and for task-based assessment of image quality (Barrett & Myers, 2003), the model of an ideal observer is successfully used in many experimental studies.

### Experiments with/without the subject’s own natural aberrations

To account for the effect of optical aberrations, the model of an ideal observer is more realistic for contrast sensitivity than for visual acuity. However, for specific studies of visual acuity, it is conceivable that subjects behave like an ideal observer. For example, studies of the effect of the subject’s natural aberrations on visual acuity with adaptive optics correction (Marcos, Sawides, Gambra, & Dorronsoro, 2008; Li et al., 2009; Legras & Rouger, 2008) may favor the model of the ideal observer that uses aberrated templates and the 25log_10_(*M*) metric of visual image quality. In the present study, subjects were not familiar with optical aberrations that were not their natural aberrations. This experimental condition may favor the model of the real observer and the 50/3log_10_|*VSOTF*| metric.

### Choice of aberrations

The approximation of theoretical observers with metrics of visual acuity (Equations 21 and 29) is the central result of our work. We have experimentally illustrated our theory with a study of aberrations that partly resembles the through-focus study of Cheng et al. (2004), which later provided the experimental data to the landmark paper of Watson and Ahumada (2008). Future work includes the test of our metrics on a wider range of aberrations.

## Conclusions

In this work, we demonstrate that the *VSOTF* and the *M* metrics relate to two models of theoretical observers that classify letters of an acuity chart using, as templates, their unaberrated and aberrated images, respectively. Our approach scales the metrics to predict changes in visual acuity due to optical aberrations, without a posteriori scale or offset. We have illustrated this theory with experiments, in which we numerically introduced combinations of defocus and spherical aberration, and pure coma. We obtained better prediction of letters lost with the 50/3log_10_|*VSOTF*| metric. Here we have used the numerical approach that directly introduces optical aberrations by convolution of the displayed images of optotypes, and the metrics can be adapted with the appropriate optical transfer functions that correspond to the actual experimental conditions. We also expect that clinical studies can benefit from using our metrics to relate aberration measurements to visual acuity changes.
